# First-in-human evaluation of [^18^F]CETO: a novel tracer for adrenocortical tumours

**DOI:** 10.1007/s00259-022-05957-9

**Published:** 2022-09-08

**Authors:** Isabella Silins, Anders Sundin, Mark Lubberink, Lleah O’Sullivan, Mark Gurnell, Franklin Aigbirhio, Morris Brown, Anders Wall, Tobias Åkerström, Sara Roslin, Per Hellman, Gunnar Antoni

**Affiliations:** 1 Department of Surgical Sciences, Uppsala University, Uppsala, Sweden; 2 Institute of Metabolic Science & Department of Medicine, University of Cambridge, Cambridge, UK; 3 Wolfson Brain Imaging Centre, Department of Clinical Neurosciences, University of Cambridge, Cambridge, UK; 4 William Harvey Heart Centre, Queen Mary University of London, London, UK; 5 Department of Medicinal Chemistry, Uppsala University, Uppsala, Sweden

**Keywords:** [^18^F]CETO, Adrenal tracer, Positron emission tomography

## Abstract

**Purpose:**

[^11^C]Metomidate positron emission tomography (PET) is currently used for staging of adrenocortical carcinoma and for lateralization in primary aldosteronism (PA). Due to the short half-life of carbon-11 and a high non-specific liver uptake of [^11^C]metomidate there is a need for improved adrenal imaging methods. In a previous pre-clinical study *para*-chloro-2-[^18^F]fluoroethyletomidate has been proven to be a specific adrenal tracer. The objective is to perform a first evaluation of *para*-chloro-2-[^18^F]fluoroethyletomidate positron emission computed tomography ([^18^F]CETO-PET/CT) in patients with adrenal tumours and healthy volunteers.

**Methods:**

Fifteen patients underwent [^18^F]CETO-PET/CT. Five healthy volunteers were recruited for test-retest analysis and three out of the five underwent additional [^15^O]water PET/CT to measure adrenal blood flow. Arterial blood sampling and tracer metabolite analysis was performed. The kinetics of [^18^F]CETO were assessed and simplified quantitative methods were validated by comparison to outcome measures of tracer kinetic analysis.

**Results:**

Uptake of [^18^F]CETO was low in the liver and high in adrenals. Initial metabolization was rapid, followed by a plateau. The kinetics of [^18^F]CETO in healthy adrenals and all adrenal pathologies, except for adrenocortical carcinoma, were best described by an irreversible single-tissue compartment model. Standardized uptake values (SUV) correlated well with the uptake rate constant *K*_1_. Both *K*_1_ and SUV were highly correlated to adrenal blood flow in healthy controls. Repeatability coefficients of *K*_1_, SUV_65–70_, and SUV_120_ were 25, 22, and 17%.

**Conclusions:**

High adrenal uptake combined with a low unspecific liver uptake suggests that ^18^F]CETO is a suitable tracer for adrenal imaging. Adrenal SUV, based on a whole-body scan at 1 h p.i., correlated well with the net uptake rate *K*_i_.

**Trial registration:**

ClinicalTrials.gov, NCT05361083 Retrospectively registered 29 April 2022. at, https://clinicaltrials.gov/ct2/show/NCT05361083

**Supplementary Information:**

The online version contains supplementary material available at 10.1007/s00259-022-05957-9.

## Introduction

The PET tracer [^11^C]metomidate ([^11^C]MTO) is an inhibitor of the enzymes 11β−hydroxylase and aldosterone synthase (CYP11B1 and CYP11B2) which are found in the adrenal cortex[1]. [^11^C]MTO selectively binds to these enzymes and is clinically used for imaging of adrenocortical pathologies, mainly adrenocortical cancer (ACC) and primary aldosteronism (PA) [2–4]. In ACC, it is used for tumour characterization, staging, and post-operative surveillance [5]. In PA, [^11^C]MTO together with dexamethasone pre-medication, is used for lateralization of the disease, primarily in patients where adrenal venous sampling (AVS) has proved unsuccessful [4]. One of the limitations of [^11^C]MTO is the short half-life of carbon-11 (20 min). A second disadvantage is the high non-specific uptake in the liver, which may confound the analysis of specific tracer binding in the right adrenal. Other possible PET tracers for adrenocortical imaging targeting CYP11B2 are [^18^F]FETO [6], [^18^F]FAMTO [7], [^18^F]CDP2230 [8] and [^18^F]AldoView [9]. To our best knowledge, only [^18^F]FETO has been clinically evaluated in a pilot study [6]. Concerns have been raised that both [^11^C]MTO and [^18^F]FETO may have radioactive metabolites with a negative impact on image quality [7].

Computed tomography (CT) can be used to detect and characterize benign adrenal adenomas [10]. However, because co-existing non-functioning adenomas may occur in PA, using CT to diagnose aldosterone-producing adenomas may render false positive results [11]. The lack of optimal imaging methods leaves adrenal vein sampling (AVS) as the current golden standard for the lateralization of PA [12]. To yield accurate results, AVS needs to be performed by experienced operators [13].

In search for an improved adrenocortical PET tracer, Erlandsson et al. investigated several fluorine-18-labelled metomidate analogues [14]. One of these, [^18^F]CETO, demonstrated high binding specificity to the adrenal cortex and favourable in vivo binding characteristics, including low non-specific uptake in the liver, compared to [^11^C]MTO [15]. Using fluorine-18, with a 110-min half-life [16], simplifies logistics in a clinical setting and extends uptake time, allowing clearance of [^18^F]CETO from the blood before the PET scan, consequently increasing the image contrast.

With all the above-mentioned in mind, we have evaluated [^18^F]CETO in healthy volunteers and in patients with various types of adrenocortical tumours.

## Methods

### Patients

Fifteen patients who had undergone clinical and imaging workup at the Department of Surgery, Uppsala University Hospital, and who harboured functioning and non-functioning adrenal tumours were informed and asked to participate in the study. Their demographical and clinical data are presented in Table 1*.*

**Table 1:** Diagnosis, location (right and/or left), size (mm), attenuation (Hounsfield units, HU) and maximum standard uptake value (SUV_max_) of [^18^F]CETO for the patients’ adrenal lesion/lesions and the healthy contralateral adrenal gland. *PA*, primary aldosteronism, *N*, normal, *Non-func*, non-functioning, *ACC*, adrenocortical cancer

Patient no.	Clinical diagnosis	Right adrenal				Left adrenal			
		Imaging diagnosis	SUV_max_	Size (mm)	Attenuation (HU)	Imaging diagnosis	SUV_max_	Size (mm)	Attenuation (HU)
1	PA	Hyperplasia	18	11 × 16	− 19	N	13		
2	Non-func adenoma	Adenoma	25	12 × 28	− 18	Adenoma	43	20 × 25	5
3	Adrenal Cushing	N	15			Adenoma	38	19 × 24	24
4	PA	Adenoma	21	7 × 8	− 5	N	24		
5	PA	N	17			Hyperplasia	14	14 × 19	− 6
6	Adrenal Cushing	N	11			Adenoma	27	19 × 25	26
7	PA	Adenoma	24	12 × 17	4	N	18		
8	PA	Adenoma	31	19 × 24	4	N	27		
9	Myelolipoma	N	15			Myelolipoma	23	27 × 27	16
10	Adrenal Cushing	Adenoma	24	21 × 28	− 5	N	24		
11	ACC recurrence	X				N	8		
12	Non-func adenoma	Calcified adenoma	33	16 × 15	78 (− 127–1444)	Calcified adenoma	24	25 × 11	148 (54–525
13	Non-func adenomas	Adenoma	28	19 × 15	− 9	Adenomas	36	13 × 24, 6 × 12	− 3, − 4
14	Non-func adenoma	Adenoma	30	16 × 26	6	N	29		
15	Non-func adenomas	Adenomas	15	10 × 17, 7 × 7	15, 4	Adenoma	12	48 × 38	− 2

Five patients with lateralized PA, according to adrenal venous sampling (AVS), were recruited from the waiting list for adrenalectomy due to a solitary adenoma or nodular, but asymmetrical, hyperplasia. Three patients diagnosed as having adrenal Cushing, with overproduction of cortisol due to primary adrenal disease were included. Five patients with non-functional adenomas were enrolled.

The two remaining patients included one with a recurrent ACC and one with an adrenal myelolipoma.

All PA patients had pathological aldosterone/renin ratio, and suspicion of PA was confirmed by the salt-loading test. When adenoma visualization was consistent with the AVS lateralization, and the adenoma was found in an otherwise normally shaped adrenal, the cause for PA was stated as an adenoma. When the adrenal gland was overall enlarged, without clear adenomatous parts, the working diagnosis was hyperplasia.

The cause of PA, such as aldosterone-producing adenoma or nodular hyperplasia, was confirmed by histopathology in 4 out of 5 patients. The remaining PA patient was diagnosed using the salt-loading test and thereafter lateralized to the right adrenal gland by AVS and is currently awaiting surgery.

All patients with adrenal Cushing were classified as “subclinical,” implying that there were no hypercortisolism-related clinical symptoms. The hypercortisolism was confirmed by the dexamethasone suppression test. The diagnosis of adrenal Cushing’s disease was confirmed by the clinical and biochemical outcome after surgery and by subsequent histopathology. The patients harbouring non-functional adrenal adenomas were diagnosed in accordance with clinical guidelines for incidentalomas [17]. The recurrence of ACC was diagnosed by histopathology. The myelolipoma was diagnosed according to typical morphological findings on CT.

Five healthy volunteers were recruited through a non-associated external provider (Clinical Trials Consultant, Uppsala, Sweden). All subjects provided written informed consent prior to inclusion. The study was approved by the Swedish Ethical Review Authority (No. 2019-00497) and the Swedish Medical Product Agency (EU No. 2018-004831-64) and registered in European Union Drug Regulating Authorities Clinical Trials Database (EudraCT no. 2018-004831-64).

### PET/CT examination

[^18^F]CETO was produced as described previously [15]. The mean and standard deviation of the administered mass of CETO was 0.76 μg (range, 0.1–1.37μg). The mean administered activity was 178 MBq (range, 89–330 MBq). There were no adverse or clinically detectable pharmacologic effects in any of the 20 subjects. No significant changes in vital signs or electrocardiograms were observed. A dynamic 90-minute PET/CT examination was performed on a Discovery MI 4 or 5 rings PET/CT scanner (General Electric, Milwaukee, Wisconsin, USA) using a 20 or 25 cm, axial field of view (FOV), respectively.

Repeated whole-body PET/CT examinations, extending from the base of the skull to the proximal thighs, were performed after the dynamic PET scan.

In the five healthy controls, the procedure was repeated after approximately 2 weeks. Prior to the dynamic [^18^F]CETO scans, three of the controls underwent a 10-min dynamic PET scan starting simultaneously with a controlled bolus injection of 400 MBq [^15^O]water.

Further details about the PET/CT examination can be found in Online Resource 1.

### Arterial blood sampling and metabolite analysis

During the first 10 min of the dynamic examination, arterial blood was drawn through a radial artery catheter by a Veenstra automatic blood sampling system for continuous whole blood measurements [18]. Arterial blood was drawn at a rate of 10 mL/minute from 0 to 3 min p.i. and 3 mL/min from 4 to 10 min p.i. Discrete blood samples were drawn from the same radial catheter at 5, 10, 15, 20, 30, 45, 60, 75, and 90 min p.i. for the determination of the percentage of intact [^18^F]CETO in arterial plasma. The whole-blood and plasma radioactivity concentrations (Bq/mL) were measured in a well counter, cross-calibrated with the PET scanner. In one patient, arterial sampling could not be performed because of technical problems. The metabolite analysis procedure has previously been described [15].

### Image-derived blood data

A Hermes hybrid viewer (Hermes Medical Solutions AB, Stockholm, Sweden) was used for image analysis. Circular 1-cm-diameter regions of interest (ROIs) were placed in the abdominal aorta in 10 consecutive slices in the frames where it was best visualized, and the ROIs were combined to a volume of interest (VOI). An image-derived whole-blood TAC was obtained by projecting the aortic VOI onto all timeframes of the dynamic PET examination.

### Arterial input curves

To achieve an arterial plasma input function, each patient’s whole-blood TAC (either sample- or image-based) was multiplied by the mean value of that patient’s plasma-to-whole-blood ratio and a sigmoid fit to intact tracer fraction over time, resulting in blood sampler-derived (BSIF) and image-derived (IDIF) input functions. Further, in addition to creating individual input curves, also IDIFs based on individual whole-blood TACs and population-averaged data for plasma-to-whole-blood ratio and parent fraction were calculated (IDIF-PA).

### Volumes of interest

Adrenal glands and adrenal tumours were outlined using 42% isocontour VOIs in a summed image of the time frames in which they were best depicted in the dynamic PET examination, (typically 22–37, corresponding to 5–80 min p.i.). VOIs were drawn manually, at approximately the same isocontour, in the subsequent whole-body PET images. Illustrations of VOI placements are found in Online Resource 3. Normal adrenals in patients were defined as the radiographically normal adrenal contralateral to the adrenal with pathologies. Representative VOIs were positioned in the other normal organs and tissues using VOIager 4.0.7 software (GE Healthcare, Uppsala, Sweden)*.* All VOIs were projected onto all time frames of the dynamic PET examination to obtain TACs. The same technique was applied in the subsequent static whole-body PET examinations to obtain data for the time-points 120, 180, and 300 min p.i. TACs were converted to standardized uptake values (SUV) by normalization to injected activity per body weight.

### Tracer kinetic analysis

An in-house developed Matlab software (The Mathworks, Natick, MA) was used for compartmental and Patlak analyses, as well as for the construction of parametric images. The operational equations of the single-tissue irreversible (1T1k), the single-tissue reversible (1T2k), the two-tissue irreversible (2T3k), and the two-tissue reversible (2T4k) plasma-input compartment models were fitted to the adrenal TAC data using weighted non-linear least-squares regression (NLR). The fitting was performed using BSIF, IDIF, and IDIF-PA as input functions. Parameter estimates of the model rate constants (*K*_1_, *k*_2_, *k*_3_, *k*_4_, and fractional blood volume *v*_A_) as well as the net uptake rate of tracer *K*_i_ = *K*_1_*k*_3_/(*k*_2_ + *k*_3_) were obtained. The optimal compartment model was chosen based on the Akaike information criterion, the Schwarz criterion, and the robustness of parameter estimates based on their standard errors [19]. The relation between IDIF and BSIF-based parameters and between IDIF and IDIF-PA-based parameters was assessed. Additionally, Patlak graphical analysis for varying time intervals (10, 20, etc.–90 min p.i.) was conducted [20]. The quantitative accuracy of *K*_i_ from the optimal plasma-input compartmental model with an IDIF was determined by comparison with the value of *K*_i_ from the optimal plasma-input compartmental model with a blood-sample-derived input function (BSIF). Furthermore, Patlak *K*_i_ values as well as *K*_i_ from the optimal compartmental model using an IDIF with patient-averaged corrections were validated against *K*_i_ from the optimal compartmental model with an IDIF. To assess the relation between SUV and outcome measures of kinetic modelling, SUV for the intervals 60–70 and 80–90 min p.i. was used. The effect of scan duration on outcome parameters was assessed by repeating the analysis using only the first 5, 15, 40, or 60 min of data.

### Parametric images

Parametric *K*_i_ images were generated using an IDIF and the Patlak method on the full 90-min dynamic PET data. Mean *K*_i_ values in the adrenals were determined using 42% isocontour VOIs in the parametric images. Parametric image *K*_i_ values were validated against *K*_i_ from the optimal compartmental model with an IDIF.

### Adrenal blood flow

VOIs drawn on the dynamic [^18^F]CETO images were transferred to the [^15^O]water scans using a Hermes hybrid viewer. Adrenal blood flow was calculated using the single-tissue compartment model, including fitted blood volume fraction, with the ascending aorta TAC as input function. The correlation between adrenal blood flow and outcome parameters of [^18^F]CETO was assessed using regression analysis.

### Test-retest analysis

Agreement between [^18^F]CETO outcome parameters of the test and retest scan was assessed using Bland-Altman analysis. Reproducibility was calculated as the mean absolute percentage difference between test and retest values, and the repeatability coefficient was calculated as 1.96 times the standard deviation of the difference between test and retest values.

### Statistics

Statistical analysis was conducted using the coefficient of determination (*R*^2^), Kruskal-Wallis test followed by Dunn’s multiple comparison, Wilcoxon and Mann-Whitney tests, orthogonal regression, and Bland-Altman analysis in GraphPad Prism version 9.1.0 (GraphPad Software, San Diego, California USA).

## Results

### Imaging

**Fig. 1:**
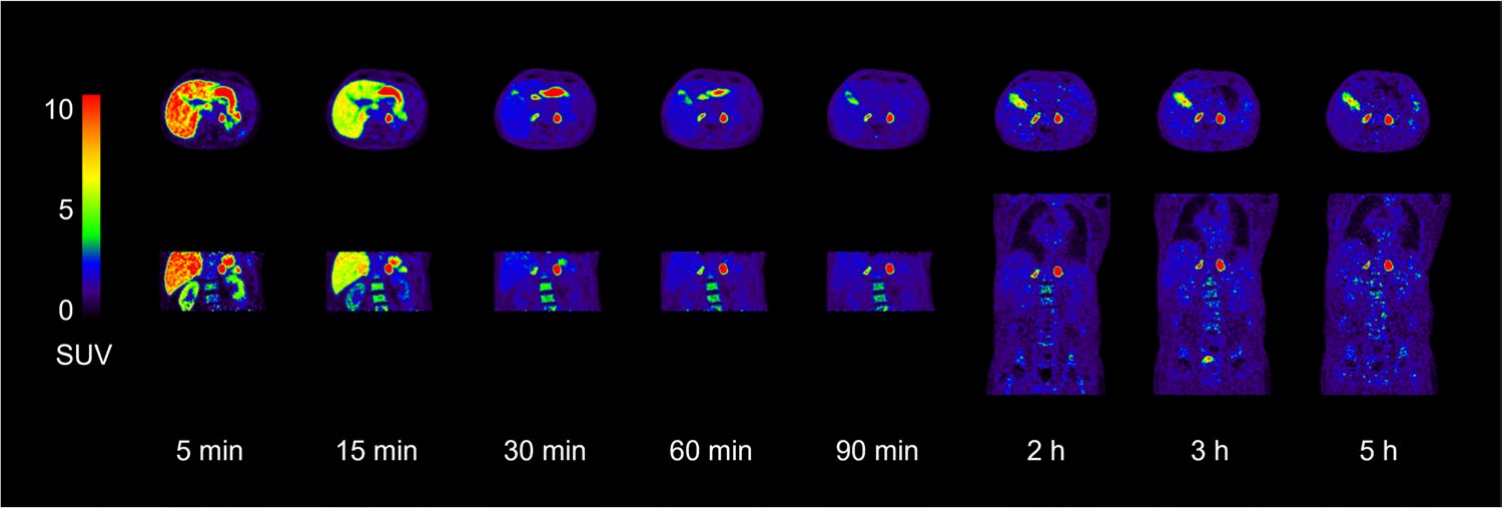
[^18^F]CETO coronal (top) and transaxial (bottom) images at 5, 15, 30 and 60 min p.i. and whole-body images at 2, 3 and 5 hours p.i.

**Fig. 2:**
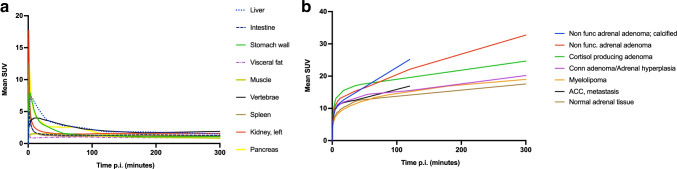
Time-activity-curves (TACs) showing the mean SUV_mean_ of [^18^F]CETO versus time post injection (x-axis). **a**: Normal organs/tissues **b**: Non-functioning adrenal adenoma, calcified), Non-functioning adrenal adenoma, Cortisol producing adenoma, aldosterone-producing adenoma/Adrenal hyperplasia, Myelolipoma, Adrenocortical carcinoma (ACC) recurrence and normal adrenal tissue. Patients with calcified non-functioning adenoma and ACC only underwent a single whole-body scan, hence no data at later time points was available

**Fig. 3:**
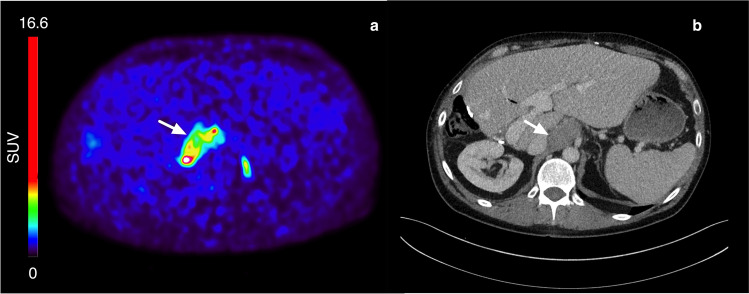
ACC metastasis(arrow), patient 11. [^18^F]CETO PET 3 hours post injection (**a**) and venous phase contrast-enhanced CT performed 17 days before [^18^F]CETO PET (**b**). A high uptake of [^18^F]CETO is seen in the ACC metastasis (**a**)

**Fig. 4:**
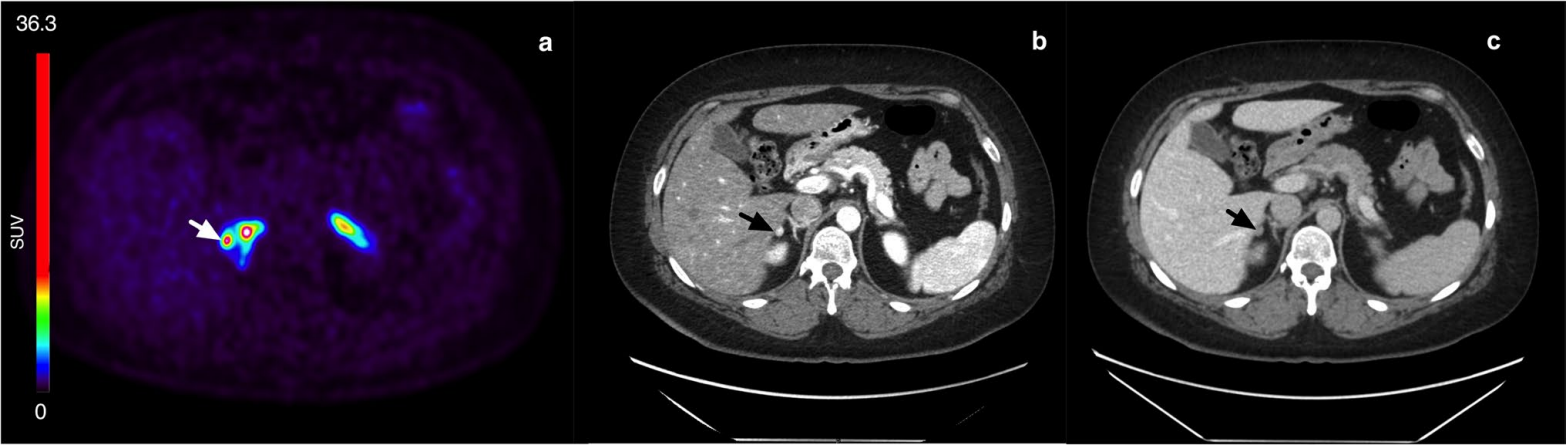
Right-sided Conn adenoma (arrow), patient 4. [^18^F]CETO PET 3 h post injection (**a**), arterial phase contrast-enhanced CT (**b**), and venous phase contrast-enhanced CT (**c**) where CT was performed 6 months before [^18^F]CETO PET

**Fig. 5:**
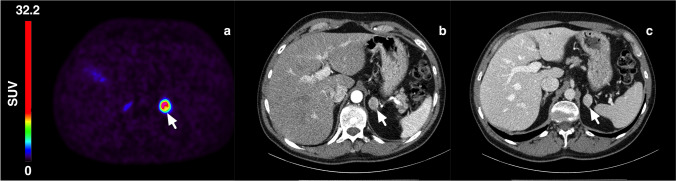
Left-sided adrenal Cushing(arrow), patient 3. [^18^F]CETO PET 3 h post injection (**a**), arterial phase contrast-enhanced CT (**b**), and venous phase contrast-enhanced CT (**c**), where CT was performed 3 days after [^18^F]CETO PET

**Fig. 6:**
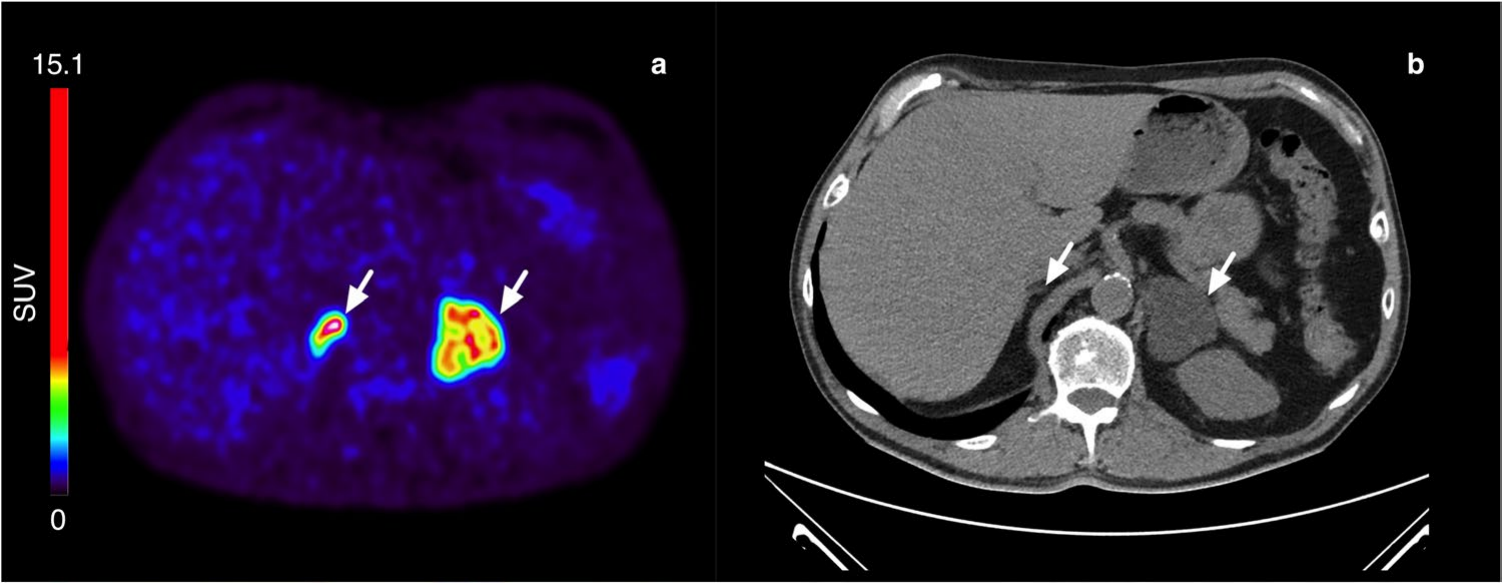
Bilateral nonfunctional adenomas(arrows), patient 15. [^18^F]CETO PET 3 h post injection (**a**) and CT (**b**), where CT was performed 17 days before [^18^F]CETO PET

Figure 1 shows typical PET images during the dynamic scan and the whole-body images at 2, 3, and 5 h p.i. [^18^F]CETO accumulated mainly in the adrenal glands and the adrenal tumours. At 30 min p.i., the uptake of [^18^F]CETO in adrenal glands and adrenal lesions was more than twice that in other organs. By the last timepoint, 300 min p.i., the adrenal mean SUV_mean_ was approximately 5 times that of other healthy organs, including the liver (adjusted p-value 0.004 liver vs. healthy adrenal gland: < 0.0001 liver vs. aldosterone-producing adenomas/adrenal hyperplasia) (Fig. 2). The highest uptake of [^18^F]CETO in healthy organs, other than adrenal glands, was seen in vertebral bodies. Individual SUV values for healthy organs from patients and healthy volunteers can be found in Online Resource 2, table 7-11. In Figs. 3, 4, 5, 6, adrenal [^18^F]CETO-PET and CT images in individual patients are shown. An ACC metastasis is illustrated in Fig. 3a, b, a right-sided Conn adenoma in Fig. 4a–c, a left-sided cortisol-producing adenoma in Fig. 5a–c, and bilateral non-functioning adenomas in Fig. 6.

### Blood sampling and metabolite analysis

**Fig. 7:**
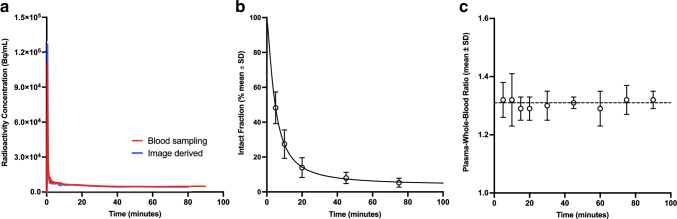
**a** Typical blood-derived and image-derived whole-blood time-activity curves, **b** mean fraction of intact tracer (*N* = 14) and **c** mean plasma-whole blood ratio (*N* = 15). The data from **a**, **b**, and **c**, multiplied together, equals the input function

Complete arterial blood data, including both the continuous on-line curve and metabolite data, were only obtained in four subjects due to technical errors in six subjects. Metabolite data was available in 14 patients, with one patient excluded since samples only could be drawn at two time points. Figure 7 shows a typical whole-blood TAC (Fig. 7a) alongside the mean fraction of the intact tracer (Fig. 7b) and mean plasma-to-whole-blood ratios (Fig. 7c) for the patients with adrenal tumours. At 5 min p.i., the mean fraction of intact tracer had decreased to approximately 50%, with approximately 10% intact tracer remaining at 45 min p.i. The mean plasma-to-whole blood ratio remained approximately constant at 1.3 throughout the duration of the scan.

### Kinetic analysis

A total of 26 (16 pathological and 10 normal) out of 30 adrenals were included in the kinetic analysis. It was not possible to conduct kinetic analysis on 2 of the 15 patients (patients 5 and 10) due to excessive motion during the dynamic scan that could not be corrected. Flowcharts for patients and healthy volunteers, visualizing which individuals/adrenals were excluded during the acquisition and analysis process, are found in Online Resource 2, Figure 1 and Figure 2. Examples of TACs for normal adrenal tissue, myelolipoma, ACC, calcifications, and adenomas fitted with all four compartmental models are shown in Online Resource (Online Resource 1 Figure 1). Generally, all different tissues showed the same behaviour except for the ACC. The adrenal affected by myelolipoma (*N* = 1), ACC (*N* = 1) and calcifications (*N* = 1) were removed from the subsequent investigation, resulting in 23 adrenals being included in the remainder of the analysis, 10 normal and 13 adenomas. Adenomas here include aldosterone-producing adenomas, adrenocortical nodular hyperplasia, cortisol-producing adenomas, and non-functional adenomas. According to the Akaike criterion, the 2T3k and 1T1k models were both preferred in 10 out of 23 cases. However, the 2T3k model generally did not produce robust parameters, with *K*_i_ equal to *K*_1_ in 10 out of 23 cases and standard errors of either *k*_2_ or *k*_3_ larger than 50% in 9 out of 13 remaining cases (Online Resource 1 Figure 2). The 1T1k model was thus chosen as the preferred model.

### Image-derived input functions

**Fig. 8:**
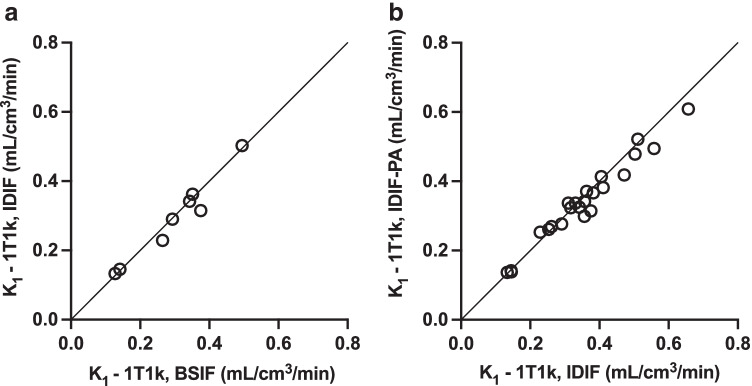
Relationship between *K*_*1*_ from the 1T1k compartment model using an IDIF (**a**) and an IDIF-PA (**b**). Black line equal to the line of identity

A strong correlation and agreement (*R*^2^ = 0.96, slope = 0.99, bias = 0.01) was demonstrated between values of *K*_1_ from the 1T1k compartment model when using an IDIF (Fig. 8a) and a BSIF (Online Resource 1 Figure 3a) for the 4 patients in which both types of input functions were available. VOI-based Patlak *K*_i_ values, determined from Patlak fits to the 10–90-, 20–90-, 30–90-, 40–90-, and 60–90-min PET images, were plotted against the 1T1k compartment model *K*_1_ values and are shown in Online Resource (Online Resource 1 Figure 3). The coefficient of determination (*R*^2^), orthogonal regression slope, and bias from Bland-Altman analysis for *K*_1_ from the 1T1k model and Patlak *K*_i_ values are listed in Online Resource (Online Resource 1 Table 2).

Validation of *K*_1_ from the 1T1k compartment model using an IDIF with patient-averaged plasma-to-whole-blood and metabolite corrections was performed by comparison against *K*_1_ from the same model but using an IDIF with patient-specific corrections (Fig. 8b). The two quantification methods demonstrated a linear relationship with strong correlation and agreement (*R*^2^ = 0.96, slope = 0.90, bias = 0.01) indicating that an IDIF-PA would be a valid alternative to an IDIF with patient-specific corrections.

An additional patient was removed from the assessment of the validation of parametric images due to a disruption in the subject’s scan, resulting in a total of 21 adrenals being included for this analysis. Parametric Patlak *K*_i_ values and values of *K*_1_ from the 1T1k compartment model showed strong correlation and agreement with one another (*R*^2^ = 0.91, slope = 1.06, bias = 0.02), in addition to exhibiting a linear relationship as seen in Online Resource (Online Resource 1 Figure 3a). A parametric Patlak *K*i image alongside its corresponding dynamic PET image is displayed in Online Resource (Online Resource 1 Figure 4). The parametric image (Online Resource 1 Figure 4b) exhibits a lower signal in the surrounding tissues in comparison with its dynamic counterpart (Online Resource 1 Figure 4a), which improves visualization of the adrenal glands.

### SUV

**Fig. 9:**
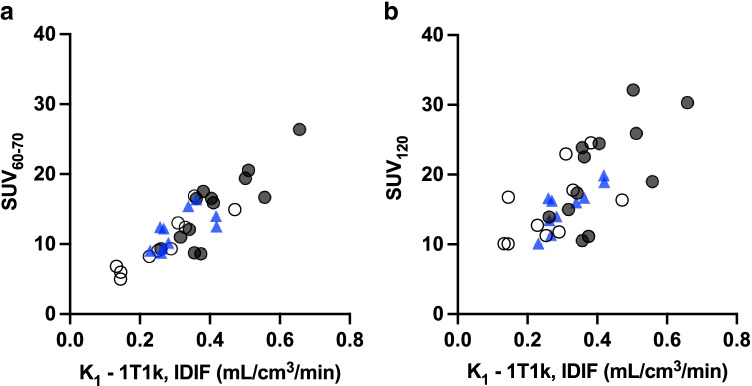
*K*_1_ from the 1T1k compartment model against SUV_60–70_ (**a**) and SUV_120_ (**b**) for adenomas (solid circles), normal adrenal tissue in patients (open circles), and healthy volunteers (blue triangles)

SUV determined at 60–70 min (SUV_60-70_) and 120 min (SUV_120_) p.i. for adenomas and normal adrenal tissue, versus *K*_1_ from the optimal compartment model is shown in Fig. 9. Moderate to high correlations were found between SUV and *K*_1_-1T1k values in adenomas (*R*^2^ 0.72 and 0.53 for SUV_60-70_ and SUV_120_, respectively), normal adrenals in patients (*R*^2^ 0.92 and 0.41), and normal adrenals in healthy controls (*R*^2^ 0.45 and 0.68). The lower correlation in healthy controls is at least in part due to the lesser variation in *K*_1_ values in this group. The relation between SUV and *K*_1_-1T1k was similar for the different tissue types.

### Effect of scan duration

*K*_1_-1T1k values based on the full 90-min scan versus corresponding values for scan durations of 5, 15, 40, and 60 min (Online Resource Figure 5a). *K*_1_ values based on shorter scan durations showed a very high correlation without significant bias down to a 5-min scan length, although with decreased precision with a maximum deviation of about 10% for a 5 min scan (Online Resource 1 Figure 5b).

### Test-retest analysis

Parent fractions in healthy controls and patients were similar, and a high agreement was found between parent fractions during test and retest scans (mean bias 0.01 ± 0.05, limits of agreement -0.08–0.11, Wilcoxon signed rank test *p* = 0.44 across all measurements at all time points). Online Resource 1 figure 6 shows correlation and Bland-Altman plots for *K*_1_-1T1k, SUV_60-70_, and SUV_120_. Retest values of *K*_1_-1T1k and SUV_60-70_ were significantly lower than test values (Wilcoxon’s test, *p* < 0.05), whereas test and retest SUV_120_ values were similar. Repeatability coefficients and absolute reproducibility were 25, 22, and 17% and 17, 16, and 7% for *K*_1_-1T1k, SUV_60–70_, and SUV_120_, respectively. In the three healthy volunteers who also underwent test-retest scans with [^15^O]water, adrenal blood flow was lower for the retest scan in 5 out of 6 adrenals (Wilcoxon’s test, *p* = 0.065).

### Adrenal blood flow

**Fig. 10:**
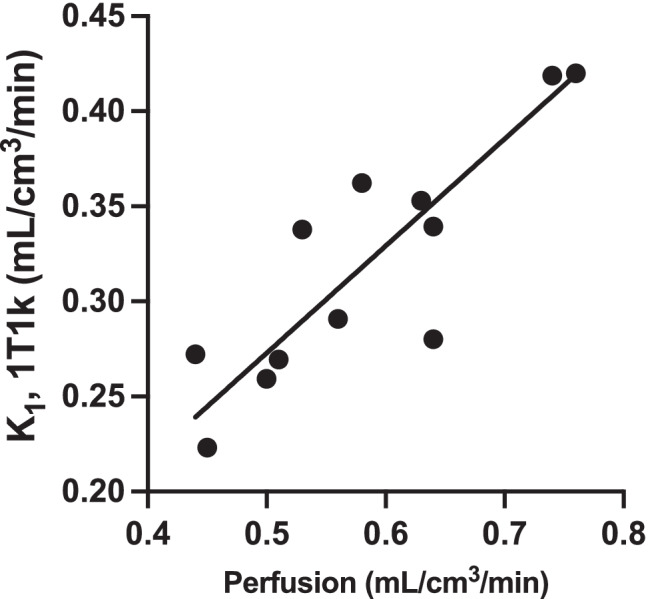
[^*18*^F]CETO *K*_*1*_-1T1k versus blood flow in the adrenal gland. Test and retest values in both adrenals are included. The solid line is a linear regression

Mean ± SD adrenal blood flow (6 adrenals in 3 patients) was not significantly different for test and retest scans, 0.62 ± 0.11 mL/cm^3^/min and 0.54 ± 0.09 mL/cm^3^/min, respectively (*p* = 0.065, Wilcoxon’s test). A significant correlation was found between adrenal blood flow and [^18^F]CETO *K*_1_-1T1k, *K*_1_-2T3k and *K*_i_-2T3k (*R*^2^ 0.73, *p* = 0.0004; *R*^2^ 0.60, *p* = 0.003 and *R*^2^ 0.57, *p* = 0.005 across both adrenals and test and retest data, respectively). Figure 10 shows the relation between [^18^F]CETO *K*_1_-1T1k and adrenal blood flow.

### Differentiation between groups

**Fig. 11:**
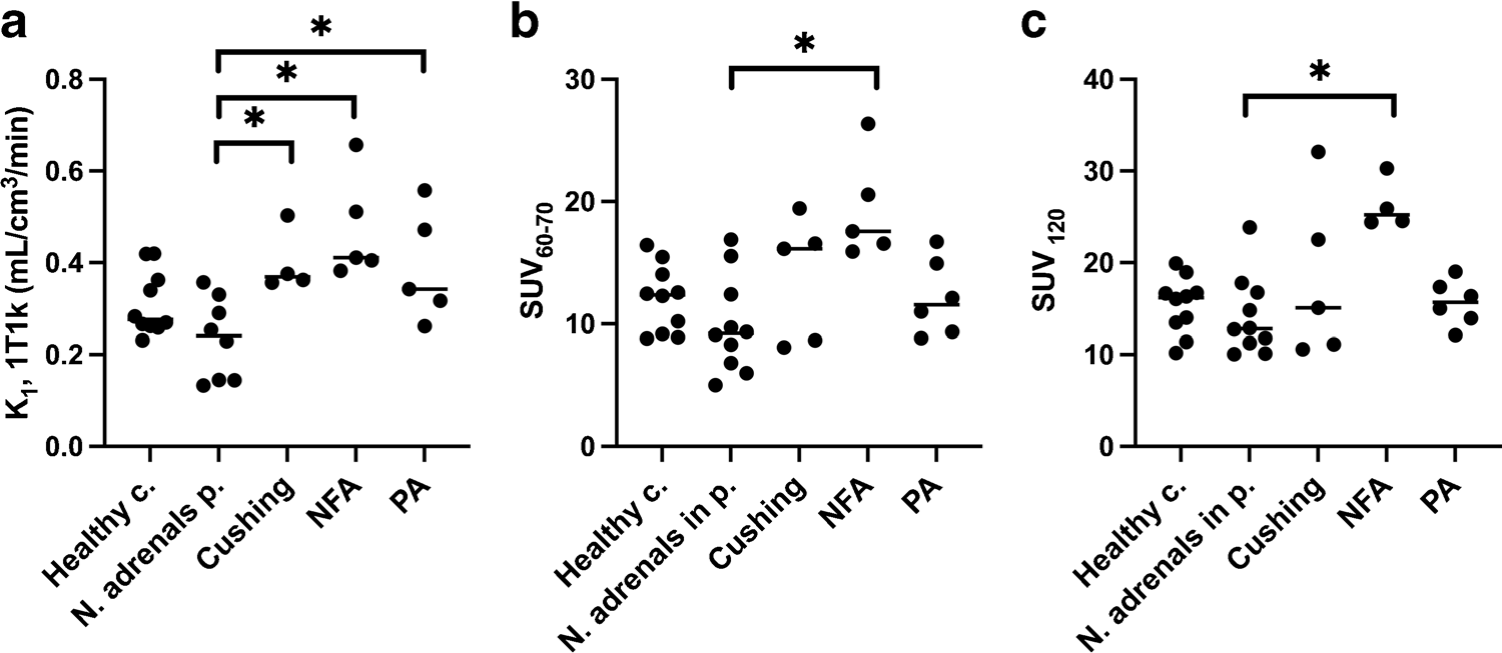
*K*_*1*_-1T1k (**a**), SUV_60–70_ (**b**), and SUV_120_ (**c**) in healthy controls, normal adrenals in patients, Cushing, NFA and PA. c. controls, n. normal, p. patients The asterisk denotes significant difference (Mann-Whitney test)

Figure 11 shows *K*_1_-1T1k (A), SUV_60–70_ (B), and SUV_120_ (C) values for adrenals in healthy controls and normal adrenals in patients (adrenal Cushing’s, NFA, and PA). Mean values are summarized in Table 2. Significant differences in *K*_1_-1T1k values were found between normal adrenals in patients and adrenals in patients with adrenal Cushing, NFA, and PA, whereas SUV only showed significant differences between normal adrenals and NFA irrespective of the time interval*.*

**Table 2:** Mean ± SD K_1_-1T1k, SUV_60-70_ and SUV_120_ values in disease groups

	*N*	*K*_1_-1T1k (mL/cm^3^/min)	SUV_60–70_	SUV_120_
Healthy controls	10	0.31 ± 0.07	12.0 ± 2.7	15.4 ± 3.1
Normal adrenals in patients	10	0.24 ± 0.09 (*N* = 8)	9.9 ± 3.9	14.2 ± 4.3
Cushing	4	0.40 ± 0.07	13.8 ± 5.1	18.8 ± 9.1
NFA	5	0.47 ± 0.11	19.4 ± 4.3	26.3 ± 2.7 (*N* = 4)
PA	6	0.39 ± 0.12 (*N* = 5)	12.2 ± 3.1	15.6 ± 2.5

## Discussion

In this first-in-human trial, we have evaluated [^18^F]CETO in a group of patients with various adrenal diseases as well as in healthy subjects. We found a low uptake of [^18^F]CETO in the liver which will facilitate the clinical assessment of pathology in the right adrenal gland. Especially in patients with ACC, liver metastases are expected to be easier to visualize and to rule out on PET/CT with [^18^F]CETO as compared to [^11^C]MTO. Here, the optimal imaging timepoint after [^18^F]CETO injection remains to be established to achieve the optimal liver metastasis-to-liver contrast. Because of the high lesion-to-background contrast ratio, ranging from 2 to 5 at 30 to 300 min after injection, [^18^F]CETO potentially also provides better visualization of smaller lesions compared to [^11^C]MTO.

The apparent relatively high uptake in the vertebrae was not likely a result of defluorination of [^18^F]CETO, because no free fluoride was observed in the metabolite analysis and defluorination would also have resulted in an overall increased uptake in bone, not just in the vertebrae. As the uptake in the vertebrae was rapidly followed by a slow decrease, neither it is likely to be explained by radiolabeled metabolites, since these would instead have resulted in a slow increase in uptake over time. Because the vertebral uptake was of the same magnitude as that in blood, the vertebra accumulation is therefore most likely reflecting the blood radioactivity in the bone marrow.

For lateralization in PA, [^11^C]MTO-PET/CT is currently performed following dexamethasone pre-treatment to increase the contrast between a potential aldosterone-producing adenoma and the normal adrenal cortex. In the present study, aldosterone-producing adenomas were seen without prior dexamethasone suppression. However, prior dexamethasone suppression might improve the quality of the [^18^F]CETO PET/CT images, something that is currently being investigated in another study [21]. Results from that study will also be compared to [^11^C]MTO-PET/CT [22].

Reliable PET-modelling requires dynamic imaging and an input function based on arterial blood sampling and correction for tracer metabolites, which is not compatible with routine clinical investigations. In four patients we compared BSIF with IDIF, which showed good correlation and consequently implying that arterial blood sampling is not required. Furthermore, the metabolite analyses indicated that a population-based metabolite correction would be sufficient for most applications. Several kinetic models were investigated and 1T1k was found to provide the best fit for [^18^F]CETO. In addition, the difference in *K*_1_ was significant between healthy adrenals and adrenal Cushing, PA, and NFA. Therefore, *K*_1_ measurements could potentially be of clinical value. Since our data supports that a 5-min static scan at 60 min p.i. might be sufficient to measure *K*_1_, dynamic imaging would not be required for clinical purposes. We compared *K*_1_-1T1k with static imaging at 60–70 min p.i. and around 120 min p.i. The calculated SUV_60–70_ and SUV_120_ showed only significant differences between normal adrenals and NFA. However, the finding that the [^18^F]CETO uptake (SUV_mean_ and SUV_max_ 0–90 min p.i.) was significantly higher in cortisol-producing adenomas than in healthy adrenals may potentially be applied as a future means to aid in the diagnosis of cortisol-producing adenomas.

In the irreversible two-tissue compartment model (2T3k), the rate constant *k*_3_ is assumed to be related to enzyme activity, whereas *K*_1_ and *k*_2_ are measures of the plasma to tissue transport and clearance from tissue, respectively. Since *k*_3_ >> *k*_2_ it cannot be measured reliably, and *K*_i_ is essentially equal to *K*_1_. This means that the uptake of [^18^F]CETO is flow limited. Figure 10 also shows a strong correlation between [^18^F]CETO *K*_1_ and adrenal blood flow, further suggesting that *K*_1_ is rather a measure of flow than of enzyme activity. However, we have previously shown in a non-human primate that the [^18^F]CETO signal can be blocked with cold etomidate [15]. Hence, even though *K*_1_ is the only parameter needed to describe the kinetics of [^18^F]CETO, it may be a combined measure of flow and enzyme activity. Further studies are needed to investigate if [^18^F]CETO is similarly flow-dependent when administered to patients with adrenal pathologies compared to healthy volunteers, since patients with adrenal Cushing’s or aldosterone-producing adenomas are suspected to have higher expression of CYP11B1 and CYP11B2 enzymes than healthy volunteers.

Retest values of *K*_1_, flow, and SUV_60–70_, but not SUV_120_, were lower than the initial test values. One hypothesis could be that stress levels were lower during the retest scan since the subjects were already familiar with the PET examination procedure, which is also suggested by the lower retest adrenal blood flow values in the three healthy controls that underwent test-retest scans with [^15^O]water. Adrenal blood flow has previously been shown to increase during severe stress [23]. This, however, does not explain why SUV_120_ values did not differ significantly, and hence, showed better reproducibility. Repeatability coefficients, which are not affected by a bias between test and retest scans, were also better for SUV_120_ than for SUV_60–70_.

Our study has several limitations. The group of study patients was small, and the blood flow measurements were merely performed in three healthy subjects. Full data for the arterial input function was only obtained in a small number of patients. Patient movement during the PET/CT examinations proved to be another obstacle when applying the ROIs and VOIs in the subsequent analysis. The strengths of the study include our thorough characterization of [^18^F]CETO including dynamic and whole-body PET, extensive modelling, test-retest, and metabolite analysis. Included healthy volunteers and patients were well characterized and the patients displayed a variety of relevant adrenal pathologies.

## Conclusion

Our study has shown the following characteristics of [^18^F]CETO: a high specific uptake in adrenal glands and a very low unspecific liver uptake. Data suggests that using IDIF would be sufficient if dynamic scanning in a clinical setting would be considered. SUV_60–70_ and SUV_120_ correlated well with the net uptake rate *K*_i_. Adrenal [^18^F]CETO uptake was flow limited in healthy controls and further studies are needed to investigate the flow dependence of adrenal [^18^F]CETO uptake in patients with adrenal pathologies

## Supplementary Information

ESM 1 (PDF 735 kb)

ESM 2 (XLSX 287 kb)

ESM 3 (PDF 6786 kb)
