# Supplementary material for: First-in-human evaluation of [18F]CETO: a novel tracer for adrenocortical tumours
Source: Eur J Nucl Med Mol Imaging. 2022 Sep 8;50(2):398–409. doi: 10.1007/s00259-022-05957-9 (PMC9816205; doi:10.1007/s00259-022-05957-9)
Supplement: Supplementary file 1 — (PDF 735 kb) [file 259_2022_5957_MOESM1_ESM.pdf]

First-in-human evaluation of [^{18}F]CETO; a novel tracer for adrenocortical tumours

Online Resource 1

Isabella Silins[1], Anders Sundin[1], Mark Lubberink[1], Lleah O'Sullivan[1], Mark Gurnell[4], Franklin Aigbirhio[6], Morris Brown[5], Anders Wall[1], Tobias Åkerström[1], Sara Roslin[2], Per Hellman[1] *, Gunnar Antoni[2]*

Departments of [1] Surgical Sciences, [2] Medicinal Chemistry and [3] Medical Sciences at Uppsala University, [4] Institute of Metabolic Science & Department of Medicine, University of Cambridge, [5]William Harvey Heart Centre, Queen Mary University of London and [6]Wolfson Brain Imaging Centre, Department of Clinical Neurosciences, University of Cambridge.

*Contributed equally as last author

First/corresponding author:
Isabella Silins, Phd-student Uppsala University

isabella.silins@surgsci.uu.se

+4673 680 73 93

ORCID ID 0000-0003-3802-0974

Detailed objectives

Can *para*-chloro-2-[^{18}F]fluoroethyletomidate positron emission computed tomography ([^{18}F]CETO-PET/CT) be used in diagnostics of adrenal tumors? Are the biochemical/pharmacological states conditions in humans with various illnesses, compared to healthy humans, such as the radio tracer is suitable?

PET/CT

The patients underwent a low-dose attenuation CT examination (120 kV, Auto mA, 10-100 mA, 0.5 s rotation time, full spiral, 3.75 mm slice thickness, pitch 1.53) in end expiration breath hold, followed by a 90-min dynamic PET examination of the upper abdomen, including the adrenal glands. Simultaneously, [^{18}F]CETO (mean 2.3 MBq/kg, range 1.3-3 MBq/kg) was injected using an automatic injector (MedRad; 10 mL tracer solution at 0.8 mL/s followed by 30 mL saline at 2 mL/s). The PET data was normalized and corrected for dead time, random coincidences, physical decay, scatter and attenuation based on the low-dose CT. The PET images were reconstructed using Time-of-Flight (TOF) Ordered Subset Expectation Maximization (OSEM) (3 iterations, 16 subsets) including resolution recovery and applying a 5 mm post-processing filter. The 90 min dynamic examination was divided into 37 timeframes with increasing lengths (1x10 s, 8x5 s, 4x10 s, 2x15 s, 3x20 s, 4x30 s, 5x60 s, 4x300 s, 6x600 s).

The acquisition time for the whole-body PET/CT examinations was 2, 3 and 4 minutes per bed-position at 120- (n=15), 180- (n=15), and 300-minutes (n=9) post injection (p.i.), respectively. The PET data was normalized and corrected for dead time, random coincidences, physical decay, scatter and attenuation based on the low-dose CT. The PET images were reconstructed as described above.

Same injection protocol, as described above, was used for injection of [^{15}O]water. Images from the dynamic [^{15}O]water PET scans were reconstructed as described above into 27 frames with durations of 1x10, 8x5, 4x10, 2x15, 3x20, 4x30 and 5x60 s.

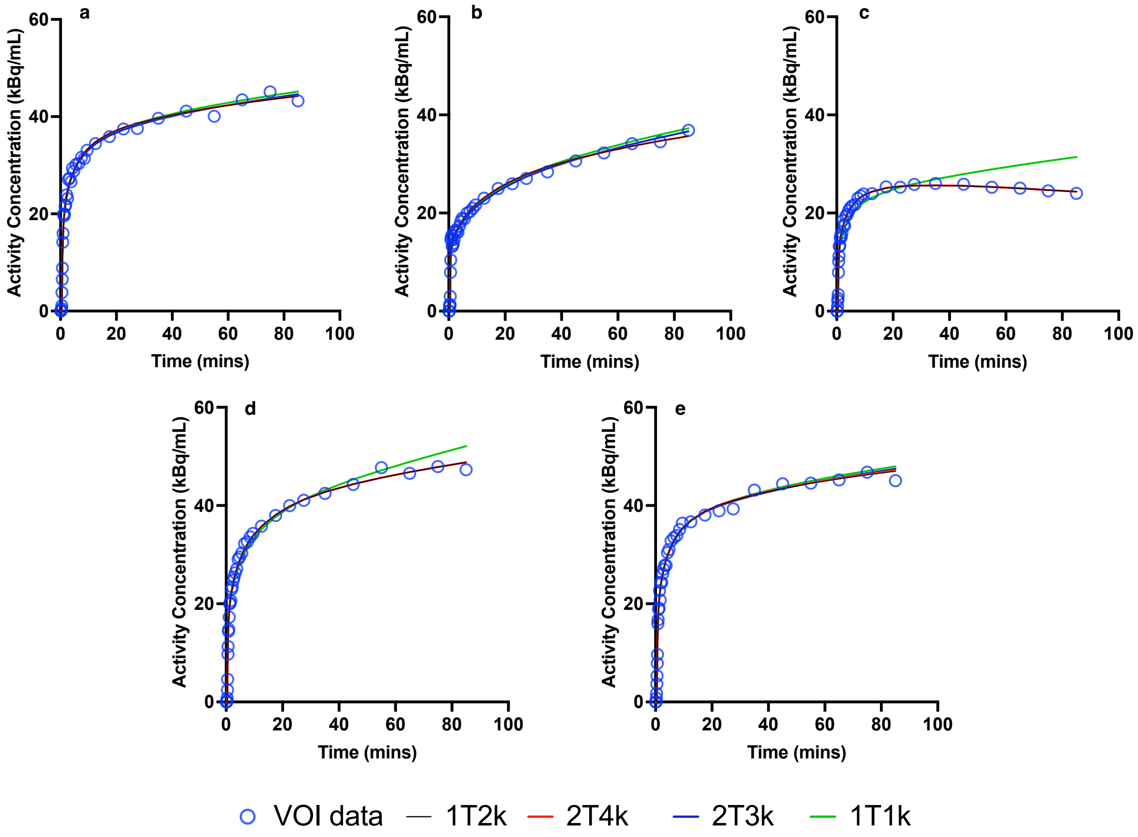

Fig. 1 Examples of time-activity curved and fits in (a) normal adrenal tissue (b) myelolipoma (c) adrenal cortical carcinoma (d) calcification and (e) adenoma. The line for each fitted model is hard to see, due to overlapping, since all models had a similar fit

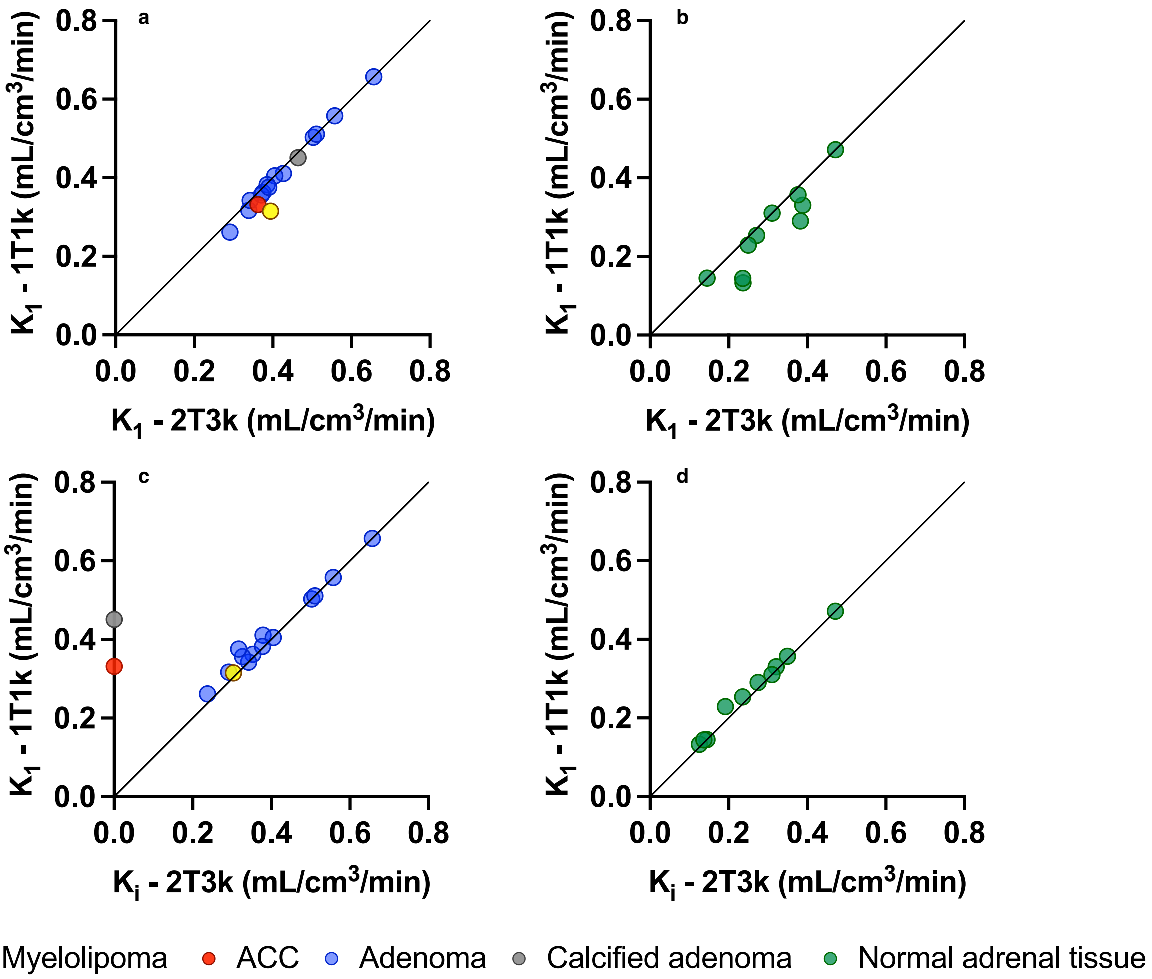

Fig. 2 Relationship between K_1 from the 1T1k compartment model using an IDIF and (a) K_1 from the 2T3k model for unhealthy adrenals (b) K_1 from the 2T3k model for normal adrenals (c) K_i from the 2T3k model for unhealthy adrenals and (d) K_i from the 2T3k model for normal adrenals. Black line equal to line of identity

Table 1 Statistical parameters between Patlak Ki and 1T1k compartment model K1 values derived using an IDIF. Brackets indicate 95% confidence intervals (slope) and 95% limits of agreement (bias).

	Acquired PET data (mins p.i)				
	10-90	20-90	30-90	40-90	60-90
R^2	0.96	0.95	0.91	0.94	0.87
slope	1.10 (0.95-1.26)	1.14 (0.93-1.34)	1.25 (0.93-1.57)	1.13 (0.96-1.29)	1.15 (0.96-1.29)
bias	0.00 (-0.06-0.06)	0.00 (-0.07-0.07)	0.00 (-0.11-0.10)	0.02 (-0.06-0.09)	0.03 (-0.07-0.12)

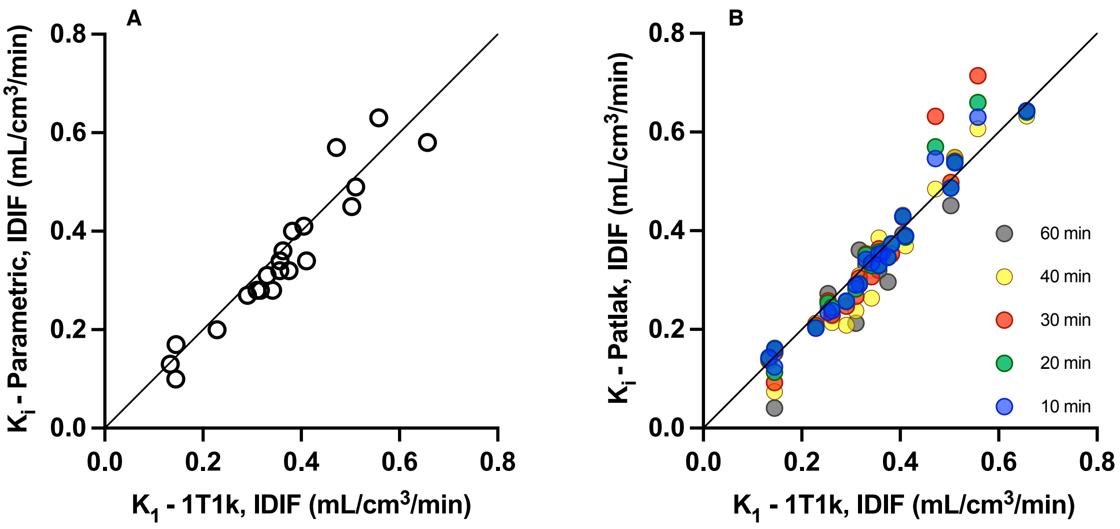

Fig. 3 Relationship between K_1 from the 1T1k compartment model using a BSIF (A) Parametric Patlak K_i values. Black line equal to line of identity (B) VOI based Patlak K_i values for varying time intervals

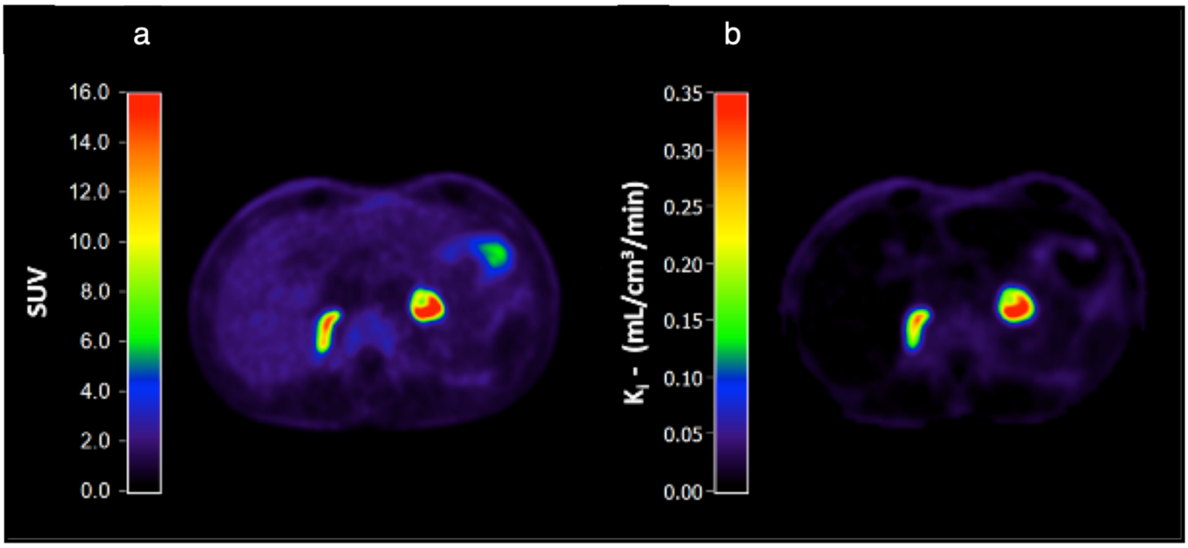

Fig. 4 Visualization of uptake of [18F]CETO in one patient (a) at 60-70 mins p.i and (b) in a parametric Patlak Ki image

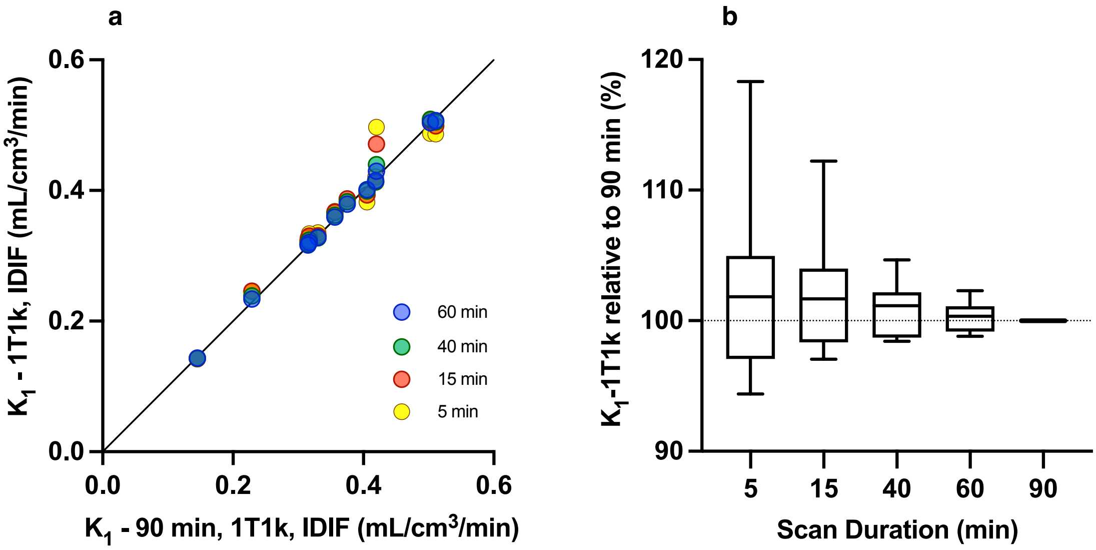

Fig. 5 K_1 values based on 5-, 15-, 40- and 60-minutes scan duration versus those based on 90 min scan duration (a) and normalized values compared to the 90 min values (b)

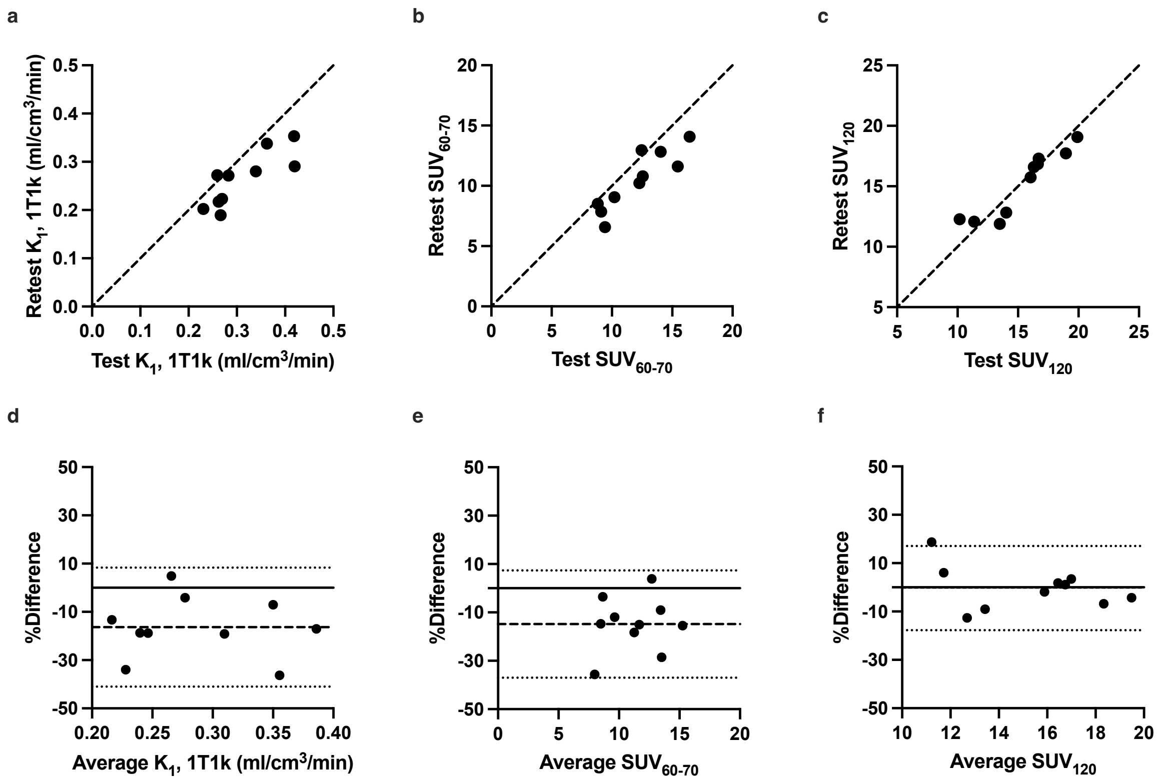

Fig. 6 Scatter (a-c) and Bland-Altman plots (d-f) of the relation between test and retest values for K_1-1T1k (A,D), $SUV_{60\text{-}70}$ (B,E) and SUV_{120} (E,F). The dashed lines in a-c are lines of identity, whereas the dashed and dotted lines in d-f denote mean bias and limits of agreement, respectively.
