# Supplementary material for: First-in-human evaluation of [18F]CETO: a novel tracer for adrenocortical tumours
Source: Eur J Nucl Med Mol Imaging. 2022 Sep 8;50(2):398–409. doi: 10.1007/s00259-022-05957-9 (PMC9816205; doi:10.1007/s00259-022-05957-9)
Supplement: Supplementary file 3 — (PDF 6786 kb) [file 259_2022_5957_MOESM3_ESM.pdf]

First-in-human evaluation of [^{18}F]CETO; a novel tracer for adrenocortical tumours

Online Resource 3

Isabella Silins[1], Anders Sundin[1], Mark Lubberink[1], Lleah O'Sullivan[1], Mark Gurnell[4], Franklin Aigbirhio[6], Morris Brown[5], Anders Wall[1], Tobias Åkerström[1], Sara Roslin[2], Per Hellman[1] *, Gunnar Antoni[2]*

Departments of [1] Surgical Sciences, [2] Medicinal Chemistry and [3] Medical Sciences at Uppsala University, [4] Institute of Metabolic Science & Department of Medicine, University of Cambridge, [5]William Harvey Heart Centre, Queen Mary University of London and [6]Wolfson Brain Imaging Centre, Department of Clinical Neurosciences, University of Cambridge.

*Contributed equally as last author

First/corresponding author:
Isabella Silins, Phd-student Uppsala University

isabella.silins@surgsci.uu.se

+4673 680 73 93

ORCID ID 0000-0003-3802-0974

DELINEATION OF VOIS IN THE DYNAMICAL SCANS CETO

<table>
<tr><td colspan="3">PET images overlaid on CT images. For delineation of VOIS with obvious and high PET-uptake, the color (Sokoloff) scale was set so that the peak level of the structure was presented as white, whereupon the delineations were done at the yellow border of the uptake. VOIager 4.0.7 (GE Healthcare, Uppsala, Sweden) was used for delineation of healthy organs/tissues. Hermes Hybrid Viewer (Hermes Medical Solutions AB, Stockholm, Sweden)) was used for delineation of aorta and adrenals.</td></tr>
<tr><td>Region
Example of region name</td><td>Slices</td><td>Standardized method for delineation</td></tr>
<tr><td>Adrenal sinister/dexter</td><td>x</td><td>The whole adrenal, was delineated all image plans. R = right adrenal, L = left adrenal

For patient 11, lacking right adrenal due to ACC, the VOI for the ACC metastasis is marked T.</td></tr>
<tr><td>Patient 3. Left adrenal: cortisol producing adenoma
Right adrenal: Normal.</td><td colspan="2">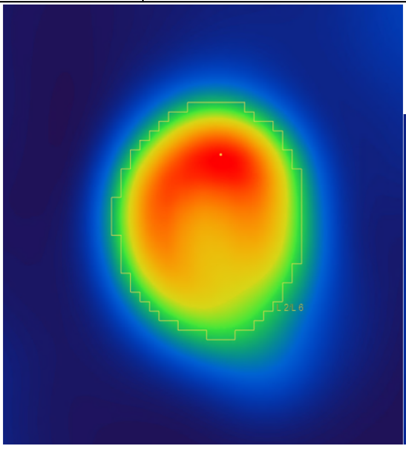

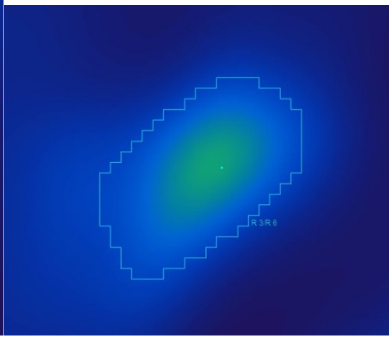
</td></tr>
<tr><td>Patient 4: Left adrenal: Normal
Right adrenal: Conn adenoma</td><td colspan="2">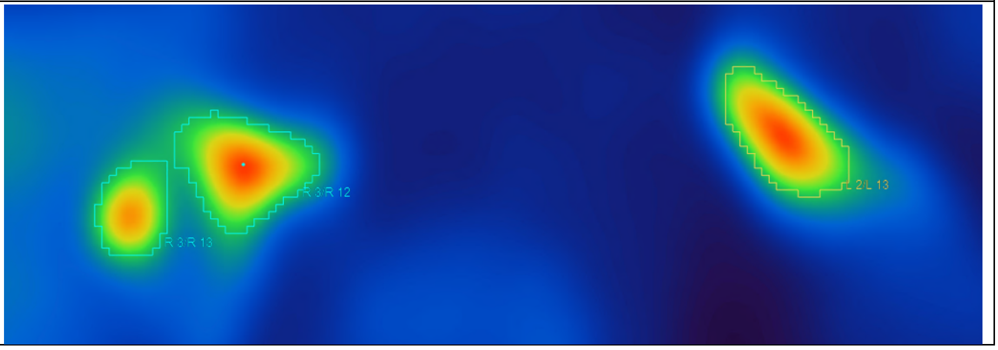
</td></tr>
</table>

<table>
<tr><td>Patient 11:
Left adrenal: Normal
Right adrenal: X
Tumor = ACC metastasis

(Red circular VOI: aorta)</td><td colspan="2">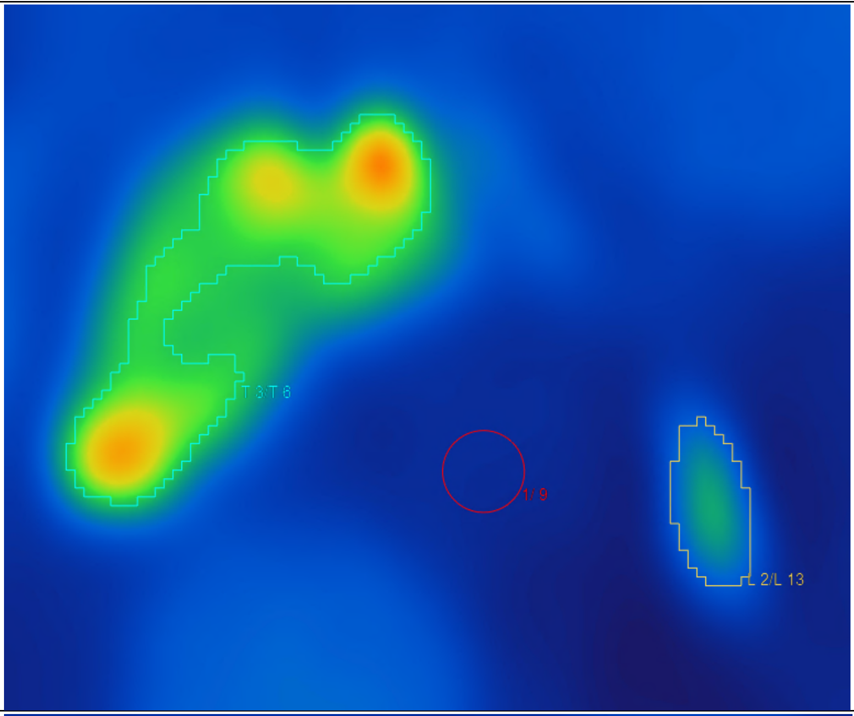
</td></tr>
<tr><td>Patient 15:
Right adrenal: Non func. Adenomas.
Left adrenal: Non func. Adenoma.</td><td colspan="2">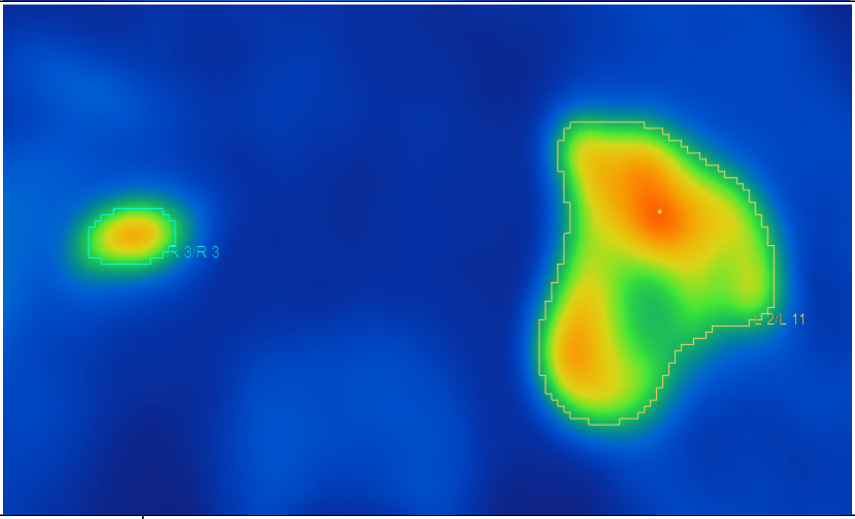
</td></tr>
<tr><td>Aorta (whole blood)
Aorta_dyn_001</td><td>5 consec. Slices PET/CT</td><td>The VOIs were delineated in Aorta-Descendens, in 5 consecutive slices, covering 4 central pixels

Anatomical landmark: first slice was located five image slices below the level where the bronchi enter the lung. Delineated in five consecutive slices downwards.
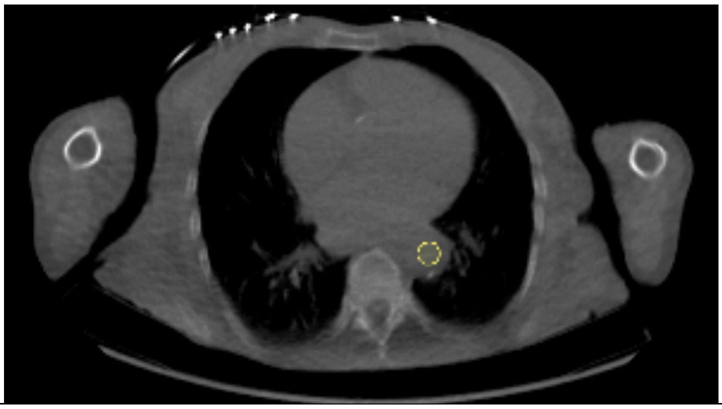</td></tr>
</table>

Spleen<br>*spleen_dyn_001*		3 consecutive and central image plans<br>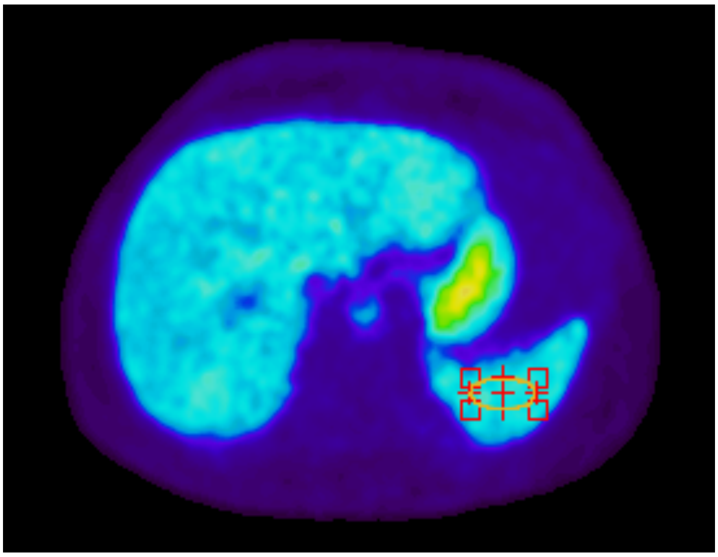
Pancreas<br>*Pancreas_dyn_003*		Representative image at upper-mid-low level,<br>Do not mix up with duodenum, (duodenum is located approx. on the kidney level and passes just frontal to aorta… Check CT image as well to see the structure, central in image plan and banana-shaped<br>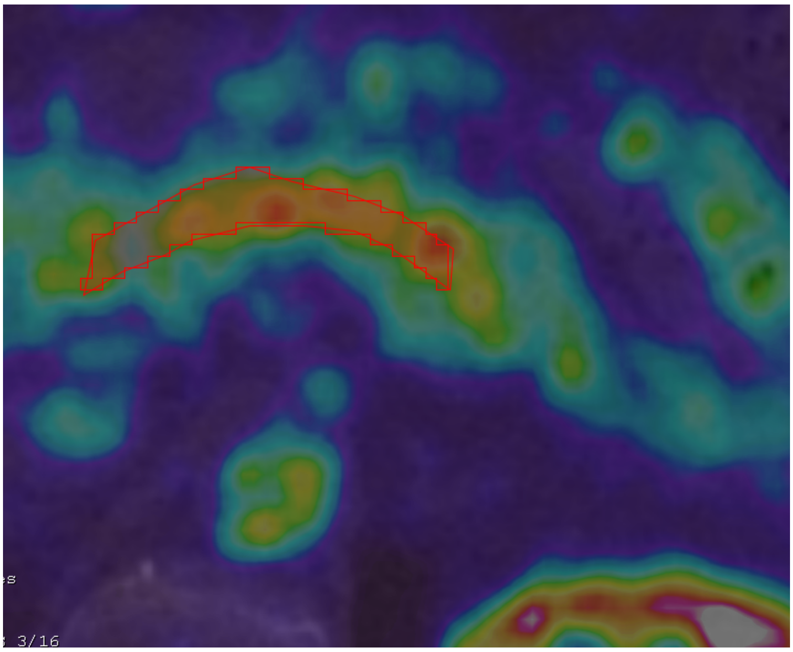
Vertebra<br>*Vertebra_001_dyn*<br>*If possible, 3 slices central in the vertebral body*<br>(Muscle VOI is visible in lower left quadrant)	3 consec. vertebrae PET/CT	Anatomical landmark: *One spinal vertebra at the levels of kidney*

<table>
<tr><td></td><td></td><td>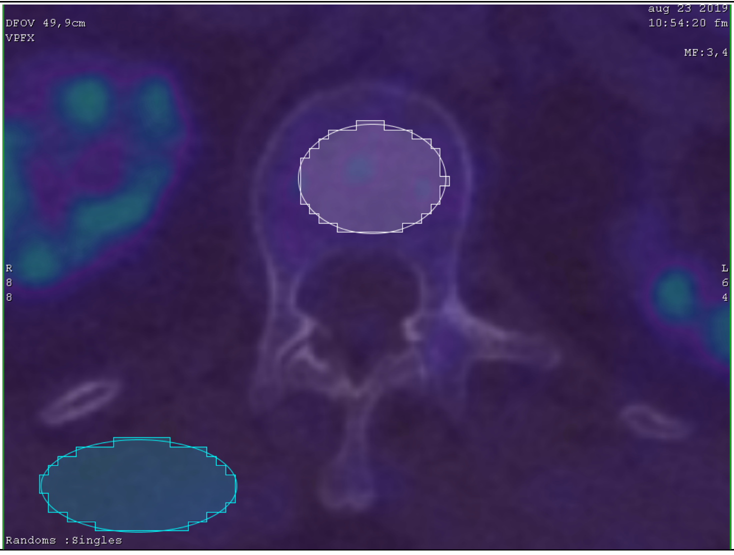
</td></tr>
<tr><td>Liver
Liver_dyn_001</td><td>1 repr.
Slice
PET/CT</td><td>One circular VOI in 3 slices, dorsal lobe (small enough to avoid partial volume effects)
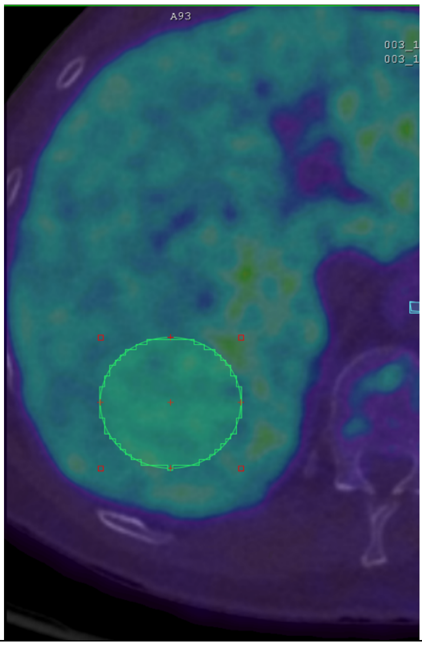
</td></tr>
<tr><td>Kidney_cortex_sin
Kideny_ctx_dyn_001_sin</td><td>3 repr.
Slice
PET/CT</td><td>3 central slices
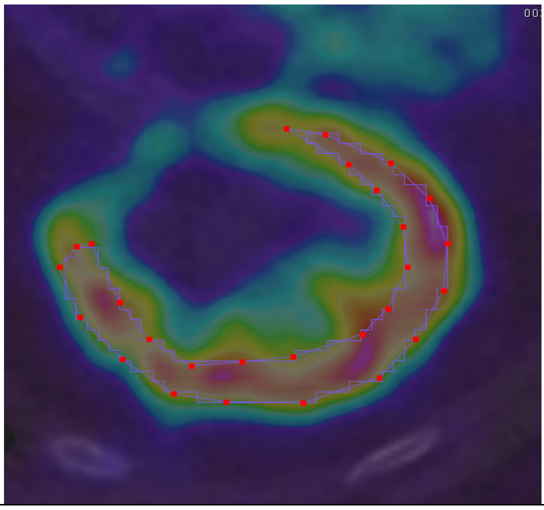</td></tr>
<tr><td>Muscle</td><td></td><td>3 slices lateral of spinal column</td></tr>
</table>

Muscle_dyn_001		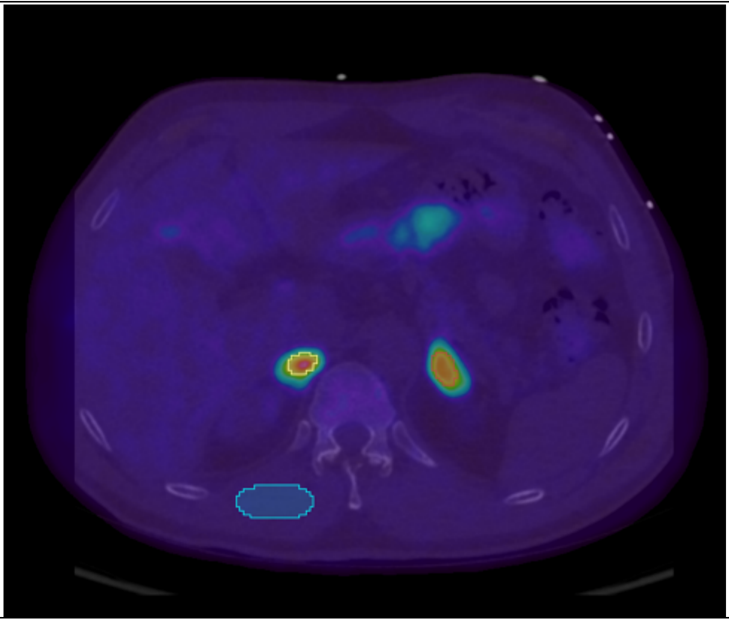
Fat<br>*Vicsc_fat_dyn_001*		3 slices below sin kidney, if not possible to mark in area above the left kidney.<br>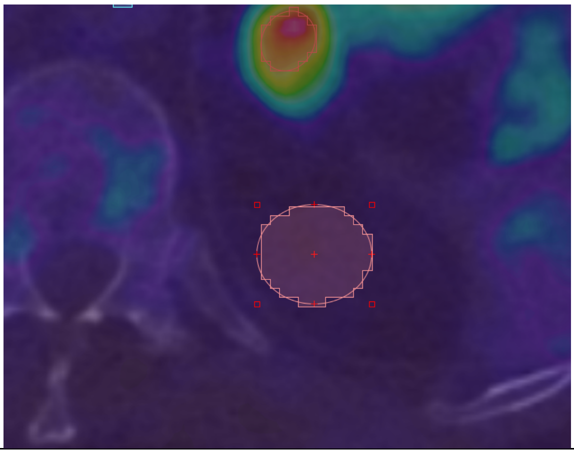
Stomach Wall<br>*Stomach_wall_dyn_001*		3 slices as a cortical roi of the stomach wall<br>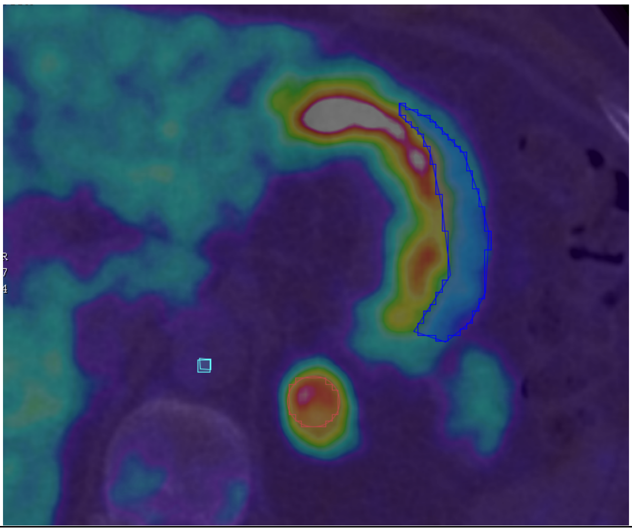

Intestine<br>*Intestine_dyn_001*		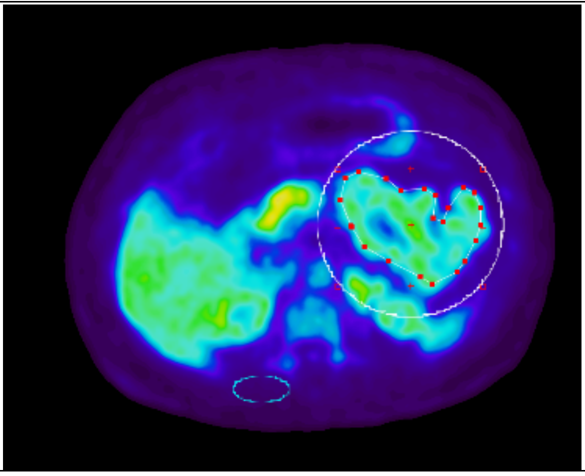
